# Effects of Saignée and Bentonite Treatment on Phenolic Compounds of Marquette Red Wines

**DOI:** 10.3390/molecules27113482

**Published:** 2022-05-28

**Authors:** Yiliang Cheng, Aude A. Watrelot

**Affiliations:** Department of Food Science and Human Nutrition, Iowa State University, 536 Farm House Lane, Ames, IA 50011, USA; ycheng8@iastate.edu

**Keywords:** color stability, fining, juice removal, interspecific cold-hardy grape, tannin

## Abstract

To improve the phenolic extraction and color stability of red wine made from cold-hardy grapes, two winemaking practices, saignée and bentonite, were applied separately and in combination on Marquette grapes at crushing. The effects of these winemaking strategies on Marquette wine’s basic chemical properties, monomeric and polymeric phenolic compounds were studied, as well as the development of color characteristics from crushing to 5 months of aging. The saignée (9% juice run-off) treatment showed little impact on the phenolic content of the finished wine, but showed an increase in color intensity. A hue shift towards an orange-yellow tone was observed in the bentonite-treated wines, which was associated with a loss of monomeric anthocyanins. The combination of saignée and bentonite showed less impact on removing anthocyanins and wine color, and increased phenolics content, therefore improving the extraction of non-anthocyanins monomeric phenolics. Although this combination treatment led to the highest concentration of tannin content after pressing, this difference between the control and other treatments disappeared over time. These results suggested that the interactions between tannins and other wine compounds still occur after removing proteins in Marquette wines.

## 1. Introduction

Condensed tannins and anthocyanins are the predominant polyphenols that influence the chemistry, sensory and quality of red wines. Condensed tannins are mainly located in the grape skins and seeds and are oligomers and polymers of flavan-3-ols, including (−)-epicatechin, (+)-catechin, (−)-epigallocatechin, and (−)-epicatechin-3-*O*-gallate [1]. As a result of the interaction between condensed tannins and salivary proteins, red wines tend to be astringent. This mouthfeel is closely associated with both the concentration and composition of tannins in red wines that are extracted during the winemaking process [2]. Anthocyanins are responsible for the red-purple color of red wines and are found mainly in grape skin and sometimes in the flesh, especially in “teinturier” varieties [3]. The five main monomeric anthocyanins in *Vitis vinifera* young red wines are the glucoside forms of malvidin, cyanidin, petunidin, peonidin, and delphinidin. Diglucoside forms of these five principal anthocyanins have been reported in native American and interspecific grapes (e.g., *Vitis rupestris, Vitis riparia,* and *Vitis labrusca*) in high quantities, with a range of 500 to 6000 mg/L of anthocyanin diglucoside and about 200 to 300 mg/L of anthocyanin monoglucoside in Frontenac and Marquette varieties [3,4]. 

Depending on the structure and solubility of phenolic compounds, their extraction from grapes to wine occur at different stages. For instance, the water-soluble phenolic compounds such as anthocyanins are extracted during the first days of alcoholic fermentation when alcohol is still at a very low content. Then, the ethanol produced during alcoholic fermentation promotes the extraction of condensed tannins from skin and seed tissues [5]. Extended maceration is a winemaking process commonly used to improve the extraction of tannins from seeds, as the longer the must is in contact with wine, the more the lipid surface of seeds change, and the more tannins can be extracted. However, this technique has some drawbacks and tends to lead to grippy and bitter wines due to the structure of extracted tannins from seeds, which are small tannins with high epicatechin-3-*O*-gallate subunit content [6]. Other winemaking techniques focus on the extraction of skin tannins to provide a more appreciated astringency mouthfeel in *Vitis vinifera* red wines such as accentuated cut edges [7,8], high power sonication [9,10], and thermovinification [11,12]. However, some of these winemaking techniques have improved the phenolic compounds in interspecific hybrid cold-hardy grape wines, which are commonly made in the U.S. Midwest [4,13,14]. It has been suggested that the low concentration of condensed tannin (<100 mg/L (+)-catechin equivalents) in interspecific wines is the result of interaction with cell wall material such as proteins and polysaccharides (e.g., pectin) [15] and the low retention of tannins throughout the winemaking process. 

In order to limit the interaction between tannins and macromolecules and improve the tannin retention in cold-hardy grape wines, using bentonite during winemaking has increasingly attracted researchers’ and winemakers’ interest in red winemaking from cold-hardy grape cultivars [16,17]. Bentonite is a negatively charged fining agent used to bind to positively charged compounds, such as proteins, to clarify and reduce enzymatic oxidation [18,19]. Bentonite treatment showed the strongest effect on protein removal in Maréchal Foch wines [17] and significantly improved the oligomeric flavan-3-ols retention only in combination with the addition of enological tannins in Frontenac wines [16]. An adverse effect on color attributes due to the removal of anthocyanin-derived pigments has also been reported [16,20,21]. The use of bentonite during the winemaking process seems promising in removing proteins but may not be the only technique needed to improve the retention of tannins in those red wines. 

Saignée is a pre-fermentation method which involves the removal of a portion of juice from crushed grapes, which modifies the solid-to-juice ratio to concentrate phenolic compounds [22,23]. Since most of polyphenols accumulate in the grape skins, removing the juice with certain level or ratio would theoretically increase the concentration of water-soluble compounds in the juice. Most research on *Vitis vinifera* red wines revealed that saignée treatment led to more red hue and higher phenolic compounds extraction in different varieties, such as Cabernet Sauvignon [24,25], Shiraz [26,27], and Monastrell [28]. Considering the correlation between the quality and the concentration of phenolics in the finished wine, more pronounced effects of the saignée treatment on color stability and phenolic compounds extraction were observed with the increase in the juice run-off percentage [28,29]. However, saignée can reduce the solvent availability to dissolve phenolics [22] and have an adverse effect on the aroma compounds of Merlot wines [29]. Consequently, 10% to 30% was the most common range of juice removal that was used to treat the must, out of consideration for commercial applicability and practical aspects.

Very little is known about the impact of both methods of combining saignée with bentonite on the retention of tannins in red wines, and it has been suggested that it could be beneficial in cold-hardy red wines to concentrate those phenolic compounds and improve their retention by removing positively charged macromolecules. The objective of this study is to assess the efficiency of saignée, bentonite, and saignée combined with bentonite (“both”) treatments on the improvement of phenolics and tannins extraction and retention in cold-hardy Marquette red wines. 

## 2. Results and Discussion

### 2.1. Basic Chemical Properties

No significant difference in pH, titratable acidity (TA), tartaric acid, and malic acid was observed between saignée treated must and the control before fermentation (Table 1). Bentonite treated must showed significantly lower pH value, TA, and malic acid content. After pressing, bentonite and saignée wines showed similar pH, while saignée wines had significantly higher TA than the control and bentonite wines. Saignée plus bentonite treatment (“both”) led to a small but significant increase (3%) in wine pH. At bottling, the bentonite and both wines showed significantly higher pH than the control and saignée wine, and significantly lower TA than the saignée wine. After five months of aging, both wines had the highest pH, TA, and concentration of organic acids among all the treatments. Bentonite wine showed comparable basic chemical properties as wines with both treatments, except for the lower content in ethanol and organic acids. These results suggested that the Marquette wine after using saignée treatment (e.g., 9% removed juice) has remained relatively unchanged in terms of the basic chemical properties in the finished wines. These results were consistent with a previous study in Cabernet Sauvignon, as no significant difference was observed in basic chemical properties between saignée-treated wine (14% juice run-off) and the control after one year of aging [24]. 

### 2.2. Wine Color

**Figure 1:**
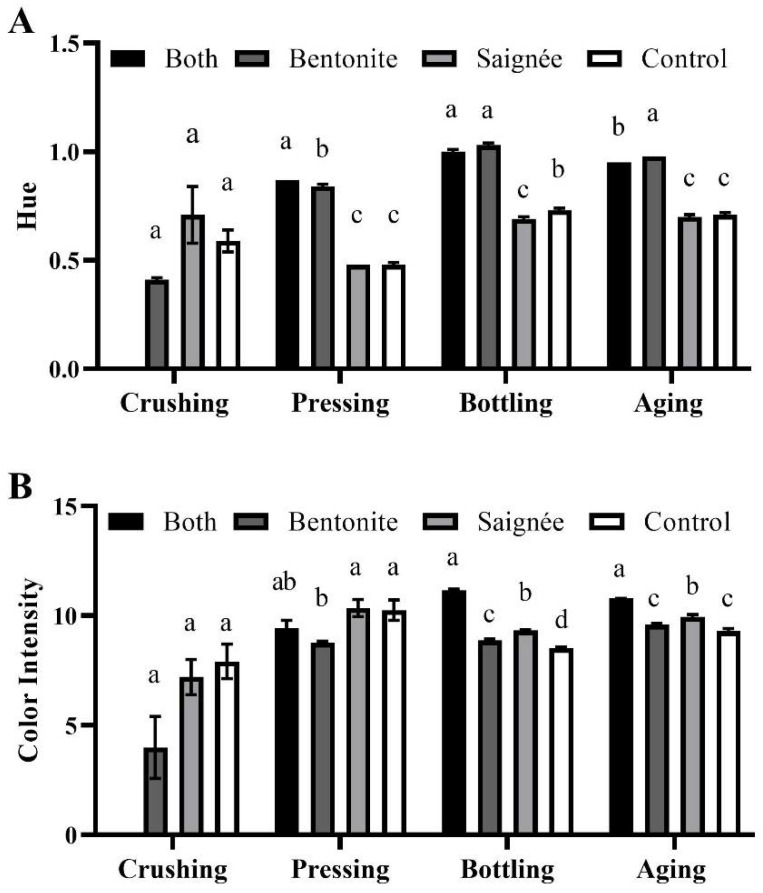
Color characteristics in Marquette grape musts and red wines throughout the winemaking process: (**A**) Hue and (**B**) Color intensity. The error bars represent standard deviation of the mean (*n* = 2). Lowercase letters indicate significant difference (*p*-value < 0.05) among treatments within same time point. “Both” means saignée plus bentonite treatment. There was no “both” treated sample collected at crushing.

Before fermentation (at crushing), no significant difference in hue and color intensity was observed between the treatments (Figure 1). After pressing, the hue of bentonite and both wines were significantly higher (79 to 85%) than the control (0.47) and saignée wines (0.48). Throughout the winemaking process, the hue increased for all the wines, but after 5 months of aging, bentonite and both treated wines still exhibited a significantly higher hue than the control (0.70) by 40 and 36%, respectively. However, these results were not correlated with the color intensity results. After pressing, the color intensity of wines was significantly higher in the control (10.25) and saignée treated wines (10.34) than the bentonite treated wines (8.77). Throughout the winemaking process, the color intensity of both treated wines increased, whereas it decreased in other treated wines. After 5 months of aging, the color intensity was significantly higher in both treated wines (10.79) than in bentonite (9.58) and control wines (9.31), being the wines with the lowest color intensity.

Saignée treated wine showed the same hue and significantly higher color intensity than the control after aging, which indicated that saignée treatment helped maintaining the red color throughout the winemaking process. This was most probably by concentrating juice, which facilitated the extraction of anthocyanins during the first days of fermentation, promoting the formation of polymeric pigments that are more stable over time [30]. The increase in hue after aging in bentonite and both treated wines indicated that the hue shifted to orange-brick, despite no significant difference in the color intensity. This might be explained by the binding affinity of bentonite to positively charged anthocyanins and, therefore, to the loss of red hue. Surprisingly, both treated wines had the highest color intensity, due to a higher absorbance at 420 and 520 nm (data not shown), which was 16% higher than the control and 13% higher than the bentonite wine after aging. This result could be explained by the reduction in anthocyanins after treatment with bentonite but the increased extraction of anthocyanins after saignée treatment. 

### 2.3. Monomeric Phenolic Compounds

The major monomeric anthocyanin in Marquette wines is malvidin-3,5-diglucoside (Table 2), which accounts for about 32% of the total anthocyanins throughout the winemaking process. This result is consistent with previous research which also states that the diglucoside form of anthocyanins accounted for 80% of the total anthocyanins in hybrid grapes [4]. Throughout the winemaking process, the total content of anthocyanins decreased over time, regardless of the applied treatment. At crushing, the content of most monomeric anthocyanins was the highest in saignée treated wines, followed by control, both, and bentonite treated wines. Both treated wines contained fewer total anthocyanins than saignée wines, but more than bentonite treated wines, regardless of the stage of winemaking. Using bentonite treatment either alone or combined with saignée significantly decreased the total concentration by 33% and 13% at bottling, and 34% and 17% after aging, respectively. This could be explained by the binding affinity of bentonite with positively charged anthocyanins, leading to their removal as suggested above. Saignée treatment increased the solid-to-juice ratio during maceration, which would likely prevent the reduction in monomeric anthocyanins content after using bentonite. It has been previously observed that using bentonite would cause a significant reduction in color intensity and anthocyanins contents in Tannat [20] and Pinot Noir [31]. When applying bentonite treatment to Tannat must during maceration, the contents of anthocyanin-derived pigments and monomeric anthocyanin decreased to 35% in wine at bottling. Regarding anthocyanin composition, González-Neves et al. [20] indicated the concentrations of delphinidin glucoside, petunidin glucoside, and acetyl glucoside had been affected more significantly by the addition of bentonite compared to other anthocyanins, which was mainly attributed to their polarity. However, in our study, no distinction of anthocyanins was observed, as the content of all monomeric anthocyanins significantly declined in bentonite treated wines. A previous study in Merlot wines showed that saignée significantly increased (~55%) the concentration of total anthocyanins when 32% of juice was removed [29]. When 14% of juice was removed from the must at crushing, the total anthocyanins content in Cabernet Sauvignon wine increased by ~ 10% and showed more red hue than the control [24]. However, in Shiraz wines, the content of total anthocyanins was not significantly different between saignée and control, no matter the amount of juice removed (8% or 17%), while the 17% juice removal showed significantly higher non-bleachable pigment and polymeric pigment content than the 8% juice removal and control [26]. 

The total non-anthocyanin monomeric phenolic compounds content increased throughout the winemaking process in control wines from 286 mg/L to 343 mg/L (Table 3). Flavan-3-ols contributed from 74% to 86% of the total non-anthocyanin monomeric phenolic compounds in aged wines, mainly represented by gallic acid, followed by (+)-catechin and (−)-epicatechin. Bentonite and both treated wines showed a significantly lower flavan-3-ols content at pressing and a significantly higher flavan-3-ols content than the control and saignée treated wines after aging, where bentonite treated wines had the highest content of (−)-epicatechin. No significant difference in most of the non-anthocyanin monomeric phenolic compounds was observed between saignée and the control, except saignée wine showed a significantly higher concentration in quercetin-3-*O*-glucoside after aging. According to Wu et al. [24], the removal of 14% juice at crushing had no significant influence on the extraction of total non-anthocyanin phenolics and flavan-3-ols in Cabernet Sauvignon wine after aging. The study of Balík et al. [32] showed that Saint Laurent red wines treated with 0.25 g/L or 1 g/L of bentonite led to a decrease in (+)-catechin and (−)-epicatechin content. The contradictory data might be due to the type of bentonite (e.g., sodium or calcium) used in the studies, which impacted the content of flavanols in treated wines differently [33].

### 2.4. Iron-Reactive Phenolic Compounds and Tannins Content

**Figure 2:**
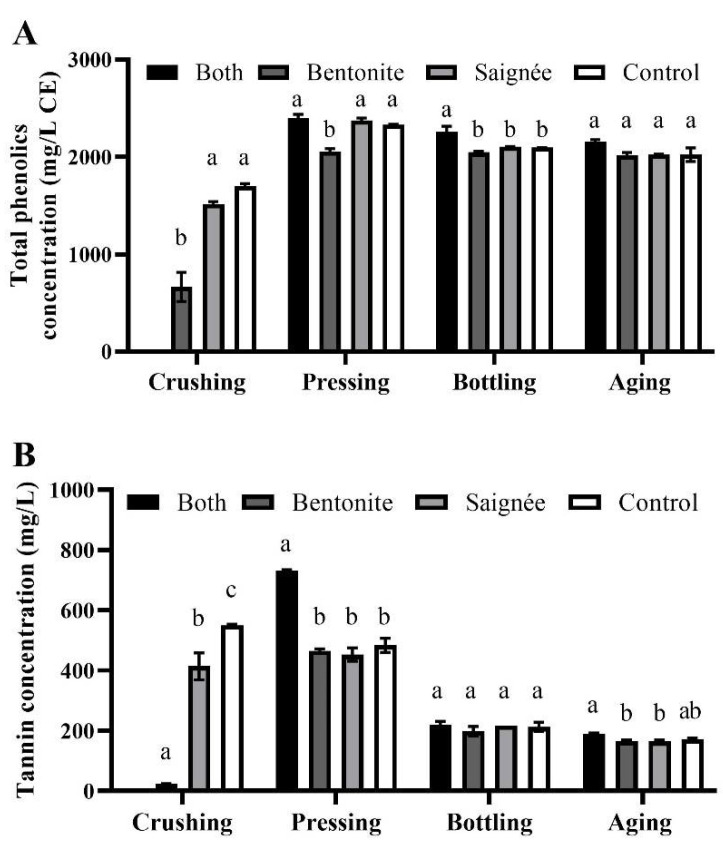
(**A**) Total iron-reactive phenolic compounds content, (+)-Catechin (CE) equivalent and (**B**) Tannin concentration in Marquette grape musts and red wines throughout the winemaking process. The error bars represent standard deviation of the mean (*n* = 2). Lowercase letters indicate significant difference (*p*-value < 0.05) among treatments within same time point. “Both” means saignée plus bentonite treatment. There was no “both” treated sample collected at crushing.

The concentration of iron-reactive phenolic compounds in control wines was much higher at 2.3 g/L (Figure 2A) than in Marquette wines from a previous study at 0.8 g/L [13], which might be attributed to the location, the year and other environmental factors of growing Marquette grapes. During the winemaking process, the content of total iron-reactive phenolics was quite stable, while the content of tannins was the highest at pressing (up to 730 mg/L) and decreased over time (up to 190 mg/L after aging) (Figure 2B). Saignée treatment had no significant effect on the concentrations of total iron-reactive phenolics and condensed tannin (Figure 2A,B). At crushing, bentonite must contained a lower concentration in both total phenolic and tannin: 2.5-fold and 23-fold lower than the control, which suggested some iron-reactive phenolics were removed by bentonite. Considering the lower juice volume (~13.7% removed) reinstated in macrobin, both-treated wines showed significantly higher tannin concentration (730 mg/L) than the bentonite treated and saignée treated sample and the control after pressing (~500 mg/L), due to the combination effect of increased solid-to-juice ratio and protein removal. However, the tannin content in both treated wines decreased more (70%) between pressing and bottling than in other treated wines (averaged 55%). This result is in agreement with previous studies, suggesting that the retention of tannins is one of the main concerns when making wine from interspecific grapes [6,13,34]. Both treatment showed a substantial extraction of tannins in Marquette wines, but this content decreased after pressing, which could be attributed to the formation of crystals of potassium bitartrate with tannins during the cold stabilization step [35]. The negligible effect of saignée treatment on tannin concentration was consistent with the study on Shiraz wine [26]. According to Casassa et al. [25], the impact of using saignée treatment to extract more tannin is restricted by multiple factors, including the solubility of tannins under different concentrations of alcohol, and further interactions with other compounds (e.g., anthocyanins) over time. The use of bentonite as a pre-fermentation process has shown 85% and 78% of protein removal in Lemberger wine (*Vitis vinifera*) and Maréchal Foch (*Vitis spp.*), but only the former showed a significantly higher content in condensed tannin at bottling [17]. The concentration of tannins only significantly increased when fermenting bentonite-treated Frontenac wines without pomace [16]. These results from bentonite-treated cold-hardy wines suggest that the interactions between condensed tannins and cell wall material still occur during maceration.

In previous studies, saignée treatment applied on Monastrell [28] and Shiraz red wine [27] showed an improvement in color intensity and a higher phenolic content, which was positively correlated with the solid to juice ratio [28]. There was no significant difference in the content of anthocyanins, tannins, and large polymeric pigments in Merlot wine treated with 16% juice run-off compared to the control. However, the content of these compounds significantly increased with the increase in juice run-off level [29]. The same conclusion was found in Shiraz wine with a higher phenolic content after 17% juice removal than after 8% juice removal [26]. In contrast with results observed in *Vitis vinifera* wines, the 9% juice run-off applied on interspecific Marquette wines in this study did not show any increase in phenolics content, probably due to the limited modification into solid-to-juice ratio compared to the control [27], or because other macromolecules limit their extraction and retention in those wines. In addition, most monomeric anthocyanins in Marquette must and wine were anthocyanin diglucoside (~80%), as shown in Table 2, which suggested that polymeric pigments were difficult to form due to the slower reaction rate between anthocyanins diglucoside and other phenolics [30].

## 3. Materials and Methods

### 3.1. Chemicals and Standards

Acetonitrile, D-fructose, ferric chloride hexahydrate, glacial acetic acid, hydrochloric acid, L-(+)-tartaric acid, *ortho*-phosphoric acid (≥85%), sodium hydroxide, sulfuric acid, and 0.1 N sodium hydroxide were purchased from Fisher Scientific (Santa Clara, CA, USA). Bovine serum albumin (BSA), caffeic acid (≥98%), (+)-catechin (≥98%), (−)-epicatechin (≥ 90%), malic acid (≥99%), quercetin-3-glucoside (≥98%), and sodium chloride were purchased from Sigma-Aldrich (St. Louis, MO, USA). Ammonium dihydrogen phosphate (>98%), potassium metabisulfite (≥97%), and sodium dodecyl sulfate were purchased from Acros Organics (Geel, Belgium). Commercial malvidin-3-*O*-glucoside chloride (oenin) (≥95%) and malvidin-3,5-*O*-diglucoside chloride (malvin) (≥95%) were provided by Extrasynthese (Genay, France). Triethanolamine was purchased from Aqua Solutions, Inc. (Deer Park, TX, USA). Milli-Q grade water (18.2 MΩ cm resistivity) used for the preparation of reagent, solutions, and high-performance liquid chromatography with diode array detector (HPLC-DAD) mobile phase was obtained from Barnstead MicroPure Water Purification System (Thermo scientific^®^, Waltham, MA, USA).

### 3.2. Winemaking Protocol

Grapes of the Vitis spp. variety Marquette (MN 1094 × Ravat 262) were harvested at the Tongue River vineyard and winery near Miles City, MT, USA on 6 September 2020, at 1347 growing degree days (GDD °C) at optimal maturity (25.5 ± 0.0° Brix, pH 3.25 ± 0.01, 10.31 ± 0.00 g/L TA, USA). After destemming and crushing, a total of 910 L of Marquette must was treated with potassium metabisulfite (35 g for 50 mg/L of free sulfur dioxide SO_2_), Scottzyme ColorPro (0.159 mL/L) Scott Laboratories, Petaluma, CA, USA), and FT Rouge (264 mg/L) (Scott Laboratories, Petaluma, CA, USA).

Four treatments were conducted as follows:

- Control: 41.6 L of must was held on skins for three days prior to yeast inoculation.
- Saignée: 41.6 L of must was held on skins for three days and 3.8 L of juice (approximate 9% *v/v* run-off) was initially removed.
- Bentonite: Must was firstly pressed to relative dryness, and 1.32 g/L sodium-calcium-bentonite fining agent (FermoBent^®^ PORE-TEC, Germany) was added into the juice. After three days, the juice was racked off the bentonite mud and fully returned (300 L) into macrobin with skins.
- Saignée plus bentonite (both): Application of the exact same protocol as the bentonite treatment, with 40 L of wine retained (13.7% *v/v* run-off) and returned into macrobin with skins after racking off the bentonite mud.

After applying the treatments, ICV D254 yeast was inoculated (264 mg/L, Scott Laboratories, Petaluma, CA, USA) with Go-Ferm (317 mg/L) (Scott Laboratories, Petaluma, CA, USA) after applying all the treatments. Malolactic bacteria (VP41) were co-inoculated after five days of alcoholic fermentation (16.5 mg/L), OptI amL (330 mg/L), Optimalo Plus (330 mg/L), and Fermaid K (198 mg/L) (all from Scott Laboratories, Petaluma, CA, USA). At the end of alcoholic fermentation (after seven days), wines were pressed, and the two bins of each treatment were blended together. After racking and cold stabilization, all of the wines received 3 g/L vanilla oak chips and 100 mg/L of free SO_2_. Two months later, all four wine treatments were bottled in 750 mL green glass bottles, flushed with argon and with 40 mg/L free SO_2_ added and closed with corks. Two samples of each treatment throughout the winemaking process (at crushing, after pressing, at bottling, and after five months of aging) were provided for chemical analysis.

### 3.3. Basic Checmial Properties

Marquette musts and wines were analyzed for TA and pH by using a digital pH meter ThermoScientific^®^ model Orion Star A211 (Waltham, MA, USA). Titration was carried out with a 0.1 N sodium hydroxide titrant to an endpoint at pH 8.20 and the results were expressed as g/L tartaric acid equivalents. The degree Brix of musts was analyzed using a digital refractometer. Organic acids, and alcohols contents in must and wines were characterized after centrifugation (accuSpin Micro 17, Thermo Fisher Scientific, Waltham, MA, USA) at 16,200× *g* for 5 min, and analysis of the supernatant by HPLC-DAD (1200 series, Agilent Technologies), using a Bio-Rad aminex HPX-87H and Bio-Rad fermentation monitoring column (column temperature: 65 °C) with H^+^ guard cartridge with 10 µL injection volume. The isocratic mobile phase, 0.005 M sulfuric acid in water, was set to a flow rate of 0.65 mL/min for 35 min. The detection of malic acid was carried out at 210 nm with a diode array detector (DAD). The detection of other organic acids and alcohols was carried out with a refractive index detector (RID) (cell temperature of 55 °C). The calibration curves of each compound were established by plotting the peak area of corresponding commercial standards from Bio-Rad versus concentration. All the must and wine samples were injected in duplicate.

### 3.4. Color Measurements

The UV/Vis absorption values of must and wines were recorded between 220 and 750 nm at 2.0 nm intervals to measure the hue and color intensity. After centrifugation at 16,200× *g* for 5 min, 350 µL of supernatant was transferred into a 1 mm pathlength Quartz cuvette (Azzota Scientific, Claymont, DE, USA) and then analyzed with a UV-Visible spectrophotometer (Genesys 150, ThermoScientific, Waltham, MA, USA). Milli-Q water was used as a blank. The hue was defined as the ratio of absorbance value at 420 nm to absorbance value at 520 nm. The color intensity was calculated as the sum of absorbance values at 420 nm-, 520 nm-, and 620 nm-times the dilution factor of 10. All the samples were evaluated in duplicate.

### 3.5. Monomeric Pheonlics Content

To determine the concentration of monomeric phenolic compounds in samples, a 1260 Infinity II HPLC (Agilent Technologies, Santa Clara, CA, USA) with a DAD (Agilent 1260 Infinity II DAD WR) and a fluorescence detector (FLD) (Agilent 1260 Infinity II FLD Spectra) was used according to the previous publications [36,37]. The mobile phases were mentioned in the previous study [38]. A reserved-phase column (LiChrospher 100-5 RP18 250 × 4.0 mm, Agilent Technologies) was used to separate the monomeric phenolic compounds at 40 °C with a flow rate of 0.5 mL/min. The injection volume for all samples was 20 µL supernatants after centrifugation at 16,200× *g* for 5 min. The monomeric phenolics were identified and quantified at different wavelengths: 280 nm for gallic acid and (+)-catechin, 316 nm for hydroxycinnamic acids, 360 nm for flavonols, and 520 nm for anthocyanins. (−)-Epicatechin was detected and quantified using the FLD, with an excitation wavelength at 276 nm and emission wavelength at 316 nm. The calibration curves were developed using (−)-epicatechin, caffeic acid, and quercetin-3-*O*-glucoside standards to quantify the concentration of flavan-3-ols, hydroxycinnamic acids, and flavonols. Anthocyanins were quantified using malvidin-3-*O*-glucoside (M3G) and malvidin-3,5-*O*-diglucoside (M35DG) standards for mono- and di-glucosides, respectively. All the samples were evaluated in duplicate.

### 3.6. Total Iron-Rective Phenolics and Tannin Content

The ferric chloride reaction based method, “Harbertson-Adams” assay, was selected to quantify the concentration of total iron-reactive phenolics and tannin in musts and wines, as previously described [39,40]. Briefly, 75 µL of the centrifuged sample was mixed with 800 µL of a resuspension buffer containing 5% triethanolamine (*v/v*) and 5% sodium dodecyl sulfate (*w/v*). The content of total iron-reactive phenolic compounds was determined based on the absorbance values at 510 nm, determined by a UV/Vis spectrophotometry (Genesys 150, ThermoScientific, Waltham, MA, USA) before and after the reaction with acidified ferric chloride reagent. The condensed tannins in must and red wines were precipitated with bovine serum albumin (BSA) and then resuspended in the buffer. The absorbance values of resuspended solution at 510 nm were recorded to calculate the differences before and after the reaction with acidified ferric chloride reagent. Both total phenolic and tannin contents were quantified and expressed as (+)-catechin equivalent (CE). The analyses were performed in duplicate for each sample.

### 3.7. Statistical Analysis

Data were expressed as the mean and standard deviation of two biological replicates and two analytical replicates. One-way analysis of variance ANOVA with the post hoc Tukey’s HSD significant difference test (α = 0.05) were conducted with the JMP^®^ Pro 16.1.0 software (SAS, Cary, NC, USA).

## 4. Conclusions

Saignée treatment showed a significantly higher wine color intensity, whereas the basic chemical properties, anthocyanin compositions and concentration, and other phenolic compounds were not much affected by the removal of 9% juice prior to fermentation. These results suggested that the effectiveness of saignée treatment on Marquette wine may need to be studied more to determine the impact of various percentage of juice run-off on wine quality. Bentonite treatment led to the removal of some phenolic compounds in Marquette wine and, consequently, caused poor color stability. The combination of saignée plus bentonite treatments (both) significantly increased the extraction of tannins during alcoholic fermentation but did not impact the retention of tannins. Both treatments on Marquette red wines improved the stability of wine color and did not impact the content of phenolics over time. However, producing Marquette red wines using the saignée method plus the addition of bentonite may not be economically feasible due to the time and work needed for limited effect on tannin and total phenolic retention. Moreover, winemakers and further study need to consider the loss of monomeric anthocyanin and the shift into yellow hue after aging when using bentonite to prevent the protein-tannin adsorption reaction.

## Figures and Tables

**Table 1:** Basic chemical properties of the Marquette grape musts and wines at four time points (crushing, pressing, bottling, 5 months aging) after different treatments. Data were expressed as mean of replicate (*n* = 2) ± standard deviation.

Time Point	Treatment	pH	TA ^3^ (g/L)	Ethanol (vol%)	Tartaric Acid (g/L)	Malic Acid (g/L)
Crushing	Bentonite	3.19 ± 0.06 b ^1^	10.03 ± 0.13 b	-	2.92 ± 0.36 a	10.52 ± 0.29 b
	Saignée	3.38 ± 0.00 a	12.84 ± 0.13 a	-	2.38 ± 0.01 a	12.54 ± 0.24 a
	Control	3.37 ± 0.01 a	14.20 ± 0.60 a	-	2.46 ± 0.10 a	12.38 ± 0.07 a
Pressing	Both ^2^	3.80 ± 0.01 a	10.50 ± 0.00 ab	-	-	-
	Bentonite	3.76 ± 0.00 ab	10.13 ± 0.26 b	-	-	-
	Saignée	3.73 ± 0.04 ab	10.79 ± 0.13 a	-	-	-
	Control	3.69 ± 0.01 b	10.13 ± 0.00 b	-	-	-
Bottling	Both	3.93 ± 0.01 a	7.13 ± 0.00 b	13.30 ± 0.39 a	4.17 ± 0.10 a	5.37 ± 0.14 ab
	Bentonite	3.91 ± 0.01 a	7.22 ± 0.13 b	13.18 ± 0.13 a	4.03 ± 0.01 a	4.69 ± 0.58 b
	Saignée	3.86 ± 0.00 b	7.50 ± 0.00 a	13.95 ± 0.39 a	3.68 ± 0.04 b	6.34 ± 0.18 a
	Control	3.85 ± 0.01 b	7.13 ± 0.00 b	14.09 ± 0.19 a	4.06 ± 0.01 a	6.44 ± 0.06 a
Aging	Both	3.74 ± 0.01 a	7.83 ± 0.07 a	14.11 ± 0.02 b	2.85 ± 0.00 a	2.75 ± 0.06 a
	Bentonite	3.70 ± 0.01 a	7.80 ± 0.03 a	13.89 ± 0.01 c	2.63 ± 0.03 c	2.42 ± 0.16 ab
	Saignée	3.63 ± 0.01 b	7.73 ± 0.07 a	14.47 ± 0.01 a	2.79 ± 0.00 b	2.30 ± 0.02 b
	Control	3.64 ± 0.02 b	7.59 ± 0.13 a	14.49 ± 0.00 a	2.81 ± 0.01 ab	2.28 ± 0.04 b

^1^ Values not connected by the same lowercase letter (a,b,c) are significantly different (*p*-value < 0.05) among treatments within same time point. ^2^ Both means saignée plus bentonite treatment. No “both” treated sample was obtained at crushing. ^3^ Titratable acidity, as tartaric acid equivalent.

**Table 2:** Monomeric anthocyanins content in Marquette grape musts and wines (in mg/L). (3-G = glucoside, 3,5-DG = diglucoside). Data were expressed as mean of replicate (*n* = 2) ± standard deviation.

Time Point	Treatment	Unknown 1	Delphinidin-3-G	Peonidin-3,5-DG	Malvidin-3,5-DG	Unknown 2	Unknown 3	Malvidin-3-G	Unknown 4	Malvidin-3-(6”-acetylglucoside)	Total Anthocyanins
Pressing	Both ^2^	78.06 ± 0.15 b ^1^	32.02 ± 0.41 b	25.30 ± 0.04 b	138.23 ± 4.90 a	25.81 ± 1.34 b	2.83 ± 0.03 a	17.20 ± 0.47 b	6.93 ± 0.36 b	5.83 ± 0.10 c	332.21 ± 6.06 c
	Bentonite	66.89 ± 0.40 c	29.96 ± 2.91 b	24.45 ± 0.51 b	123.90 ± 0.42 b	24.36 ± 0.55 b	3.00 ± 0.21 a	16.98 ± 0.56 b	6.33 ± 0.45 b	5.55 ± 0.25 c	301.42 ± 3.49 d
	Saignée	105.10 ± 1.78 a	60.73 ± 0.72 a	30.21 ± 1.65 a	145.68 ± 1.89 a	46.79 ± 1.70 a	4.53 ± 1.19 a	25.56 ± 0.11 a	14.01 ± 0.50 a	14.05 ± 0.03 a	446.66 ± 4.73 a
	Control	102.27 ± 0.21 a	57.33 ± 3.53 a	27.84 ± 0.03 ab	139.62 ± 1.38 a	42.10 ± 1.64 a	4.27 ± 0.04 a	24.92 ± 0.45 a	13.43 ± 0.11 a	12.92 ± 0.20 b	424.69 ± 4.77 b
Bottling	Both	68.79 ± 4.11 b	29.79 ± 1.36 b	13.63 ± 1.59 a	105.55 ± 5.02 a	18.65 ± 0.83 b	1.38 ± 0.20 a	12.92 ± 0.89 b	5.68 ± 0.40 b	5.42 ± 0.28 b	261.81 ± 11.51 b
	Bentonite	50.40 ± 0.58 c	22.24 ± 0.00 c	13.24 ± 0.10 a	79.82 ± 3.36 b	14.48 ± 0.08 b	1.25 ± 0.00 a	10.40 ± 0.17 b	4.43 ± 0.06 b	4.20 ± 0.07 b	200.47 ± 3.92 c
	Saignée	82.34 ± 2.21 a	44.42 ± 0.57 a	13.44 ± 2.93 a	96.95 ± 4.65 a	29.60 ± 0.31 a	1.61 ± 0.61 a	19.32 ± 0.40 a	10.94 ± 0.26 a	10.25 ± 0.73 a	308.87 ± 5.66 a
	Control	75.91 ± 0.47 ab	43.12 ± 3.38 a	15.60 ± 0.30 a	97.41 ± 0.87 a	29.48 ± 2.08 a	1.37 ± 0.10 a	17.74 ± 1.29 a	10.29 ± 1.00 a	10.17 ± 0.95 a	301.09 ± 8.90 a
Aging	Both	56.04 ± 1.67 b	21.19 ± 0.51 b	9.39 ± 0.76 b	76.70 ± 2.82 a	14.05 ± 0.37 b	0.73 ± 0.06 bc	8.51 ± 0.16 b	4.01 ± 0.04 b	4.15 ± 0.13 c	194.78 ± 6.18 b
	Bentonite	43.07 ± 0.17 c	15.79 ± 0.31 c	8.92 ± 0.05 b	61.15 ± 2.17 b	11.19 ± 0.12 c	0.71 ± 0.02 c	7.15 ± 0.13 c	2.88 ± 0.13 c	2.73 ± 0.20 d	153.60 ± 2.65 c
	Saignée	69.09 ± 1.91 a	31.77 ± 0.97 a	13.05 ± 1.14 a	81.04 ± 0.45 a	22.53 ± 0.16 a	0.85 ± 0.00 ab	13.18 ± 0.25 a	7.98 ± 0.18 a	8.25 ± 0.20 a	247.74 ± 3.86 a
	Control	64.66 ± 0.13 a	30.01 ± 0.05 a	11.86 ± 0.47 ab	76.51 ± 0.09 a	21.72 ± 0.51 a	0.90 ± 0.03 a	12.41 ± 0.34 a	7.79 ± 0.28 a	7.37 ± 0.02 b	233.25 ± 1.60 a

^1^ Values not connected by the same lowercase letter (a,b,c) are significantly different (*p*-value < 0.05) among treatments within same time point. ^2^ Both means saignée plus bentonite treatment.

**Table 3:** Monomeric phenolic compounds (non-anthocyanin) content in Marquette grape musts and wines (in mg/L). Data were expressed as mean of replicate (*n* = 2) ± standard deviation.

Time Point	Treatment	Gallic Acid	(+)-Catechin	(−)-Epicatechin	Caftaric Acid	Quercetin-3-O-glucoside	Myricetin	Quercetin	Total Non-Anthocyanin Phenolics
Pressing	Both ^2^	85.07 ± 0.03 a ^1^	87.24 ± 0.76 a	29.17 ± 0.38 b	19.82 ± 0.01 b	13.95 ± 0.61 b	0.96 ± 0.05 b	2.18 ± 0.12 b	238.38 ± 0.36 b
	Bentonite	78.59 ± 0.19 b	88.59 ± 0.23 a	27.96 ± 0.15 b	23.06 ± 0.61 b	12.65 ± 0.64 b	0.81 ± 0.05 b	2.15 ± 0.05 b	233.80 ± 1.63 b
	Saignée	66.64 ± 1.28 d	102.92 ± 8.16 a	33.90 ± 1.26 a	58.89 ± 1.60 a	18.78 ± 0.27 a	1.44 ± 0.05 a	3.13 ± 0.06 a	285.71 ± 12.02 a
	Control	70.08 ± 0.34 c	98.62 ± 0.06 a	34.28 ± 0.03 a	61.37 ± 0.11 a	17.28 ± 0.38 a	1.50 ± 0.22 a	3.18 ± 0.08 a	286.32 ± 0.20 a
Bottling	Both	134.90 ± 6.90 a	103.71 ± 8.83 a	39.34 ± 0.90 a	27.26 ± 1.27 b	15.47 ± 0.51 a	3.27 ± 0.09 a	4.64 ± 0.06 a	328.58 ± 18.57 a
	Bentonite	117.70 ± 2.94 b	99.59 ± 2.10 a	37.54 ± 1.76 a	27.71 ± 0.61 b	11.82 ± 0.11 b	2.17 ± 0.00 b	3.42 ± 0.09 b	299.95 ± 7.21 ab
	Saignée	80.98 ± 0.08 c	92.73 ± 1.00 a	32.70 ± 0.29 b	58.81 ± 1.14 a	17.35 ± 0.34 a	3.40 ± 0.01 a	4.62 ± 0.09 a	290.59 ± 0.21 ab
	Control	80.50 ± 1.31 c	90.52 ± 1.05 a	33.01 ± 0.14 b	57.19 ± 1.04 a	15.75 ± 1.03 a	3.59 ± 0.44 a	4.78 ± 0.46 a	285.33 ± 2.57 b
Aging	Both	158.66 ± 0.00 a	112.94 ± 5.85 a	51.87 ± 0.63 a	27.33 ± 0.01 b	13.24 ± 0.04 c	5.18 ± 0.20 b	6.03 ± 0.12 a	375.25 ± 6.61 a
	Bentonite	150.64 ± 0.79 b	119.43 ± 2.67 a	52.22 ± 0.10 a	29.75 ± 0.05 b	10.43 ± 0.04 d	3.66 ± 0.03 c	4.68 ± 0.02 b	370.79 ± 1.64 a
	Saignée	94.05 ± 1.21 c	117.41 ± 7.45 a	43.66 ± 0.41 b	62.67 ± 0.30 a	15.48 ± 0.05 a	5.67 ± 0.05 a	6.39 ± 0.19 a	345.34 ± 5.82 b
	Control	96.15 ± 0.82 c	115.41 ± 4.26 a	43.43 ± 0.76 b	61.59 ± 1.32 a	14.37 ± 0.50 b	5.97 ± 0.11 a	6.14 ± 0.45 a	342.80 ± 4.06 b

^1^ Values not connected by the same lowercase letter (a,b,c) are significantly different (*p*-value < 0.05) among treatments within same time point. ^2^ Both means saignée plus bentonite treatment.

## Data Availability

Not applicable.

## Sample Availability

Samples of the compounds not available from the authors.
